# Neuraminidase-specific antibody responses are generated in naive and vaccinated newborn nonhuman primates following virus infection

**DOI:** 10.1172/jci.insight.141655

**Published:** 2020-12-17

**Authors:** Patrick K. Shultz, Kali F. Crofts, Beth C. Holbrook, Martha A. Alexander-Miller

**Affiliations:** Department of Microbiology and Immunology, Wake Forest School of Medicine, Winston-Salem, North Carolina, USA.

**Keywords:** Immunology, Vaccines, Adaptive immunity, Influenza

## Abstract

Individuals younger than 6 months of age are at significant risk from influenza virus infection; however, there is currently no vaccine approved for this age group. Influenza virus neuraminidase (NA) has emerged as a potential additional target for vaccine strategies. In this study, we sought to understand the ability of newborns to mount an antibody response to NA. Here we employed a nonhuman primate model, given the similarities to humans in immune system and development. We measured antibody to NA following infection with an H1N1 virus or following vaccination and challenge. Administration of an inactivated virus vaccine was not capable of eliciting detectable NA-specific antibody, even in the presence of adjuvants previously shown to increase total virus-specific IgG. However, both naive and vaccinated newborns generated a NA-specific antibody response following virus infection. Interestingly, the presence of the vaccine-induced response did not prevent generation of systemic antibody to NA following challenge, although the respiratory response was reduced in a significant portion of newborns. These findings are the first, to our knowledge, to evaluate the newborn response to the influenza NA protein as well as the impact of previous vaccination on generation of these antibodies following virus infection.

## Graphical abstract

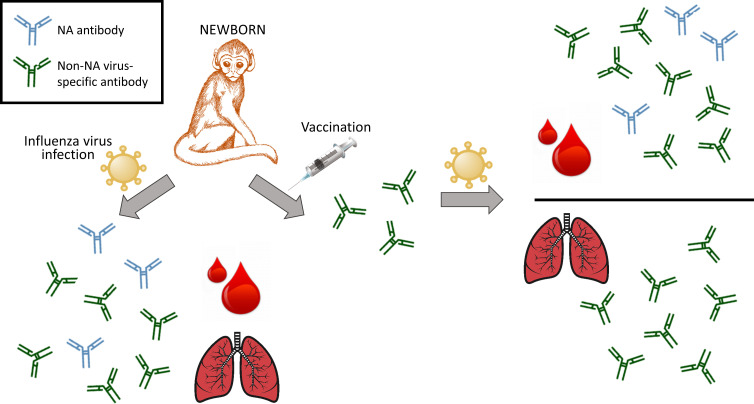

## Introduction

Neuraminidase (NA) is an influenza virus surface protein that cleaves sialic acid on the host cell surface, allowing newly formed virions to be released (1–3). In the absence of NA activity, hemagglutinin (HA) remains bound, thereby preventing virus spread. NA also functions to prevent virus aggregation by desialylating HA, increasing its infectivity (3). Finally, there is evidence that NA can contribute to viral entry through cleavage of sialic acid, allowing the virus to more efficiently penetrate the mucus layers of the respiratory tract (1, 4).

There is accumulating evidence of the potential for antibody capable of inhibiting NA enzymatic activity to contribute to viral clearance and protection (5–13). NA-inhibiting (NAI) antibody can be detected in most individuals, although the induction of these antibodies following infection is significantly reduced compared with antibodies to HA (14). This is likely due, at least in part, to the lower amounts of NA in the virion (40–50 molecules compared with 300–400 HA molecules) (15). The reduced antibody response to NA is even more apparent following administration of the seasonal inactivated vaccine, with 1% compared with 87% of influenza-specific antibodies recognizing NA versus HA (14). While inhibiting NA cannot prevent the host from becoming infected, it does limit virus spread. Thus, antibody to NA can lessen virus-related damage in the lungs in animal studies (16) and provide protection in humans (5–12). As a result, NA-specific antibodies are an attractive target in the search for a vaccine that can provide broader recognition (13, 14, 17, 18).

Infection with influenza virus poses a substantial threat for newborns and young infants (19–21). These individuals are highly susceptible to infection, with infants younger than 6 months of age carrying a significantly increased risk for severe disease resulting in hospitalization and even death compared with older children (22, 23). Influenza-associated disease is the result of severe damage and inflammation to the respiratory epithelium that can lead to pneumonia (24). The increased risk for more severe disease in young infants is the result of their naive status combined with altered immune responsiveness in this age group (25, 26). The latter is responsible for the lack of efficacy in young infants and thus the absence of an approved influenza vaccine for infants younger than 6 months.

Increasing the ability of newborns to more effectively combat influenza virus infection or respond to NA-targeted vaccines requires a deeper understanding of the capacity of such individuals to produce these antibodies and the signals that optimally promote their generation. Newborns are challenged in the ability to mount a robust antibody response following infection or vaccination (see refs. 27, 28). This is the result of multiple alterations in the immune response, including decreased DC maturation, reduced T follicular helper (Tfh) cell generation, and poorer upregulation of molecules contributing to B cell activation and differentiation (29–32). At present, we know little about how effectively newborns can generate a response to NA.

In the study presented here, we utilized a nonhuman primate (NHP) model to evaluate the ability of newborns to produce NA-specific antibody following vaccination and infection. This model was selected as it is the closest to humans with regard to immune development in early life and innate sensor distribution and function. The results from these studies provide insights into our understanding of the newborn immune response to influenza virus infection and could help inform strategies for developing a protective vaccine.

## Results

### Newborn African green monkeys produce NAI antibodies following infection with influenza A virus.

The ability of newborns to mount an antibody response capable of inhibiting NA activity following infection with influenza virus has not to our knowledge been explored. To address this question, we inoculated 4 newborn African green monkeys (AGMs; 6–10 days of age) with either 1 × 10^9^ (3 animals) or 1 × 10^8^ (1 animal) 50% egg infective doses (EID_50_) of mouse-adapted influenza A virus strain A/Puerto Rico/8/34 (H1N1) (PR8). The lower-dose animal was part of a dose escalation experiment during development of our infection model. As the response was similar to that of the animals receiving the high dose, the 4 newborns were pooled for analysis. Four adult animals (aged 6–9 years) were infected with 5 × 10^9^ EID_50_ PR8 in parallel. Blood was sampled on postinfection day 14 (d14 p.i.) and the presence of NA-specific antibodies measured. We utilized 2 approaches to evaluate responses to NA: (a) a cell-based ELISA to quantify total IgG antibody to the NA protein (33) and (b) an enzyme-linked lectin assay (ELLA) to measure antibodies with NAI activity (34). The former was important as antibodies that can bind NA in the absence of blocking enzymatic activity have been described (35).

**Figure 1:**
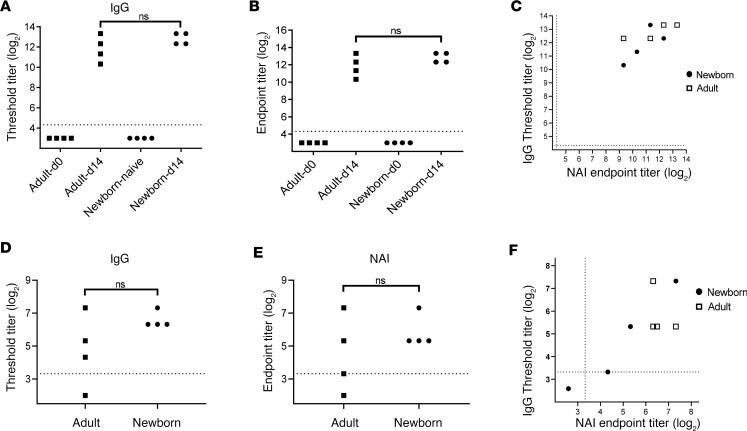
Infant AGMs generate NA-specific antibodies with inhibitory activity following infection with influenza virus. Infant (6–10 days of age) and adult AGMs (6–9 years of age) were infected with PR8 (*n* = 4/group). The presence of total NA-specific IgG (**A**) and NAI antibody (**B**) was assessed on d14 p.i. (**C**) ELLA and ELISA values for antibody in plasma from individual animals. Total NA-specific IgG (**D**) and NAI antibody (**E**) in BAL were also measured. (**F**) ELLA and ELISA values for antibody in BAL from individual animals. Preinfection samples for the adult animals used in the study are shown. The naive newborn data were obtained from a separate cohort of age-matched animals, as we did not have preinfection samples available for testing. The dotted line shows the limit of detection (LOD) for the assay. Significance was assessed using an unpaired Student’s *t* test.

Both newborn and adult animals had high levels of NA-specific IgG in the plasma on d14 following infection (Figure 1A). These animals also had antibodies with readily detectable NAI activity (Figure 1B). No significant difference in titer was detected between these groups for either NA-specific total or NAI antibody. In general there were similar trends in the 2 readouts (Figure 1C), suggesting that a similar portion of the NA-specific antibody present in adult and newborn animals had NAI activity. The comparable amounts of NA-specific IgG in the newborn and adult animals is in agreement with our previous analysis of total influenza-specific IgG antibody in these animals at this time point (36).

Given the localization of influenza virus to the lungs, we evaluated the antibody available to combat infection in this tissue. Total NA-specific (Figure 1D) and NAI antibody (Figure 1E) in bronchoalveolar lavage (BAL) fluid obtained from the newborns and adults on d14 p.i. was measured. NA-specific antibody was detected in 3 of 4 adult animals and all of the newborns (Figure 1D). Antibodies with NAI activity were similarly detected (Figure 1E). The titers for the 2 readouts for individual animals are shown in Figure 1F. While the result was not statistically significant, we noted the unexpected trend of higher antibody titers to NA in newborns, which was not evident in our previous analyses of total influenza-specific IgG antibody in the respiratory tract (36). Whether the antibody response to NA at this site may be differentially regulated compared with the overall response is not clear. These data show that newborns readily generate NAI antibodies following influenza virus infection and that these important effector molecules are present in both circulation and the respiratory tract.

### Vaccination with inactivated influenza virus does not result in detectable antibody that can recognize NA at 10 days following boost.

Having established the ability of newborns to produce NAI antibodies following infection, we next evaluated their generation in response to vaccination. We had previously assessed the influenza virus–specific response of newborn AGMs administered formalin-inactivated PR8 (IPR8) in combination with experimental adjuvants that included flagellin (a TLR5 agonist) and R848 (a TLR7/8 agonist) (37–41). For delivery of these adjuvants, flagellin was mixed with IPR8 (IPR8+flg), while R848 was conjugated to the virus particle (IPR8-R848) as described in ref. 42. We also evaluated the response generated in the presence of the combined adjuvants (IPR8-R848+flg). Vaccination with IPR8 plus an inactive flagellin (IPR8+m229) served as a non-adjuvanted control group, and PBS administration served as a non-vaccinated control. Newborns received a boost dose 21 days following initial vaccination. Given our previous finding that the presence of flagellin, R848, or the combination of the 2 adjuvants could drive increased antibody responses and viral clearance (Table 1 and refs. 40, 41), we anticipated that these adjuvants would result in higher NA-specific antibody.

Before assessing these responses, it was important to determine wether there were enzymatically active NAs in the vaccine. Activity would validate the structural integrity of the protein and thus support its potential to elicit antibodies whose recognition depended on an appropriate conformation. IPR8, IPR8-R848, heat-inactivated PR8, and nontreated PR8 were assessed for NA activity by ELLA. While treatment with formalin considerably reduced NA activity, the activity was detectable, consistent with the presence of conformationally dependent epitopes that may be required for antibody recognition (Supplemental Figure 1; supplemental material available online with this article; https://doi.org/10.1172/jci.insight.141655DS1).

**Figure 2:**
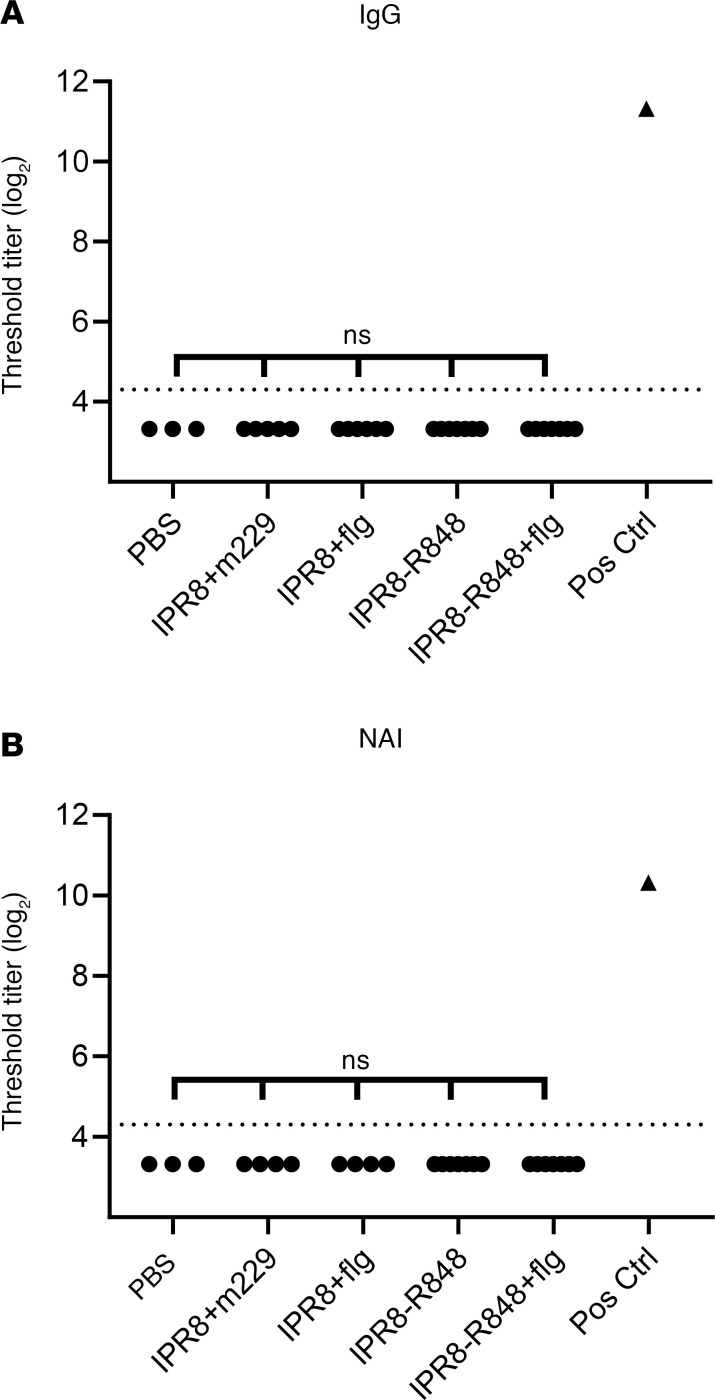
NA-specific antibodies are not detected in vaccinated infants on d10 p.b. Newborn AGMs were vaccinated with IPR8+m229, IPR8+flg, IPR8-R848, or IPR8-R848+flg or received PBS. Newborns were boosted 21 days following initial vaccination. Plasma was evaluated on d10 p.b. by ELISA for measurement of total NA-specific IgG (**A**) and by ELLA to quantify NA-inhibiting antibody (**B**). The analysis included newborns vaccinated with IPR8+m229 (*n* = 5), IPR8+flg (*n* = 6), IPR8-R848 (*n* = 7), or IPR8-R848+flg (*n* = 7). Three animals were in the control group that received PBS. The data from an infected newborn run in parallel with the assay is shown as a positive control (Pos Ctrl). The dotted line shows the LOD for the assay. Statistical analysis was performed using 1-way ANOVA with Tukey’s correction for multiple comparisons.

Antibody in the plasma was measured on post-boost d10 (d10 p.b.). Significantly elevated antibody titers to the PR8 virion resulting from the boost dose were present at this time (40, 41). No antibody to NA was detected at this time point by either the cell-based ELISA or ELLA approaches (Figure 2). These data show that vaccination with IPR8 — even in the presence of adjuvants that significantly boost the generation of total PR8-specific IgG antibodies — does not induce detectable NA-specific antibody at d10 p.b., a time at which infants had high levels of total PR8-specific IgG.

### The presence of influenza-specific antibody generated in response to vaccination does not impact the level of systemic NA antibodies following challenge.

**Figure 3:**
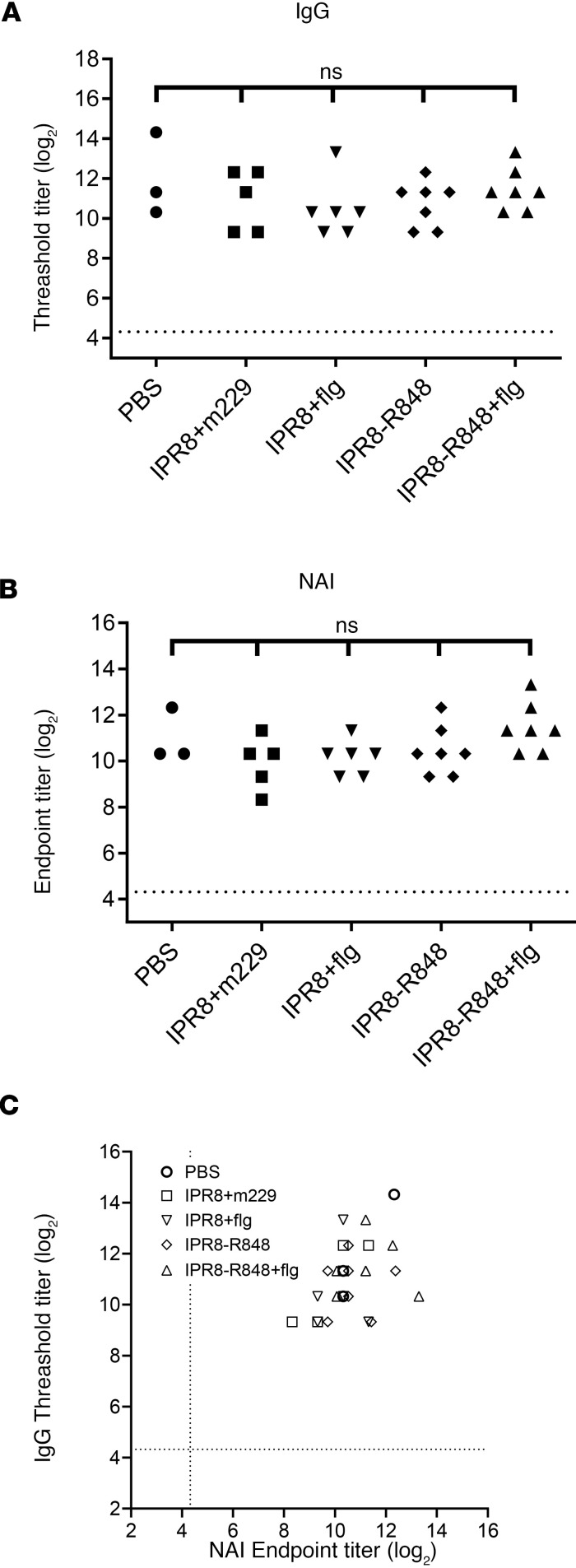
Vaccine-elicited antibody does not affect the level of NA-specific antibody generated following viral challenge. Vaccinated newborn AGMs were challenged with PR8 on days 23–26 following boost. Plasma was tested on d14 p.c. using (**A**) ELISA to measure NA-specific IgG or (**B**) ELLA to measure NA-neutralizing antibody. (**C**) ELLA and ELISA values for antibody in plasma from individual animals are shown. The dotted line shows the LOD for the assay. Statistical analysis was performed using 1-way ANOVA with Tukey’s correction for multiple comparisons.

Given the potential benefits of NAI antibodies in protecting against infection (43), we wanted to understand the effect of vaccine-elicited antibody (where antibody was directed to non-NA influenza virus proteins) on the generation of NA-specific antibody during infection. Vaccinated newborns were challenged with PR8 on day 23–26 following boost. Vaccinated and nonvaccinated (PBS-treated) newborns did not have significantly different levels of circulating NA-specific IgG (Figure 3A) or NAI antibodies (Figure 3B) on post-challenge d14 (d14 p.c.). The values from the 2 readouts for individual animals is shown in Figure 3C.

The similar level of NA-specific antibody generated across the groups is in contrast to the adjuvant-associated increase in total influenza virus–specific IgG present in these animals following challenge (Table 1 and refs. 40, 41). Thus, while we cannot rule out the possibility that this antibody response arose from reactivation of vaccine-induced, NA-specific memory B cells, we favor the hypothesis that this is a new response based on the similar levels of anti-NA antibody present in vaccinated and naive (PBS-treated) animals. Together, these data show that preexisting antibody to influenza virus proteins other than NA does not impact the circulating level of NA-specific antibody generated in newborns as a result of infection.

### Vaccinated newborn AGMs have lower respiratory NAI antibody levels than nonvaccinated animals 14 days following infection.

**Figure 4:**
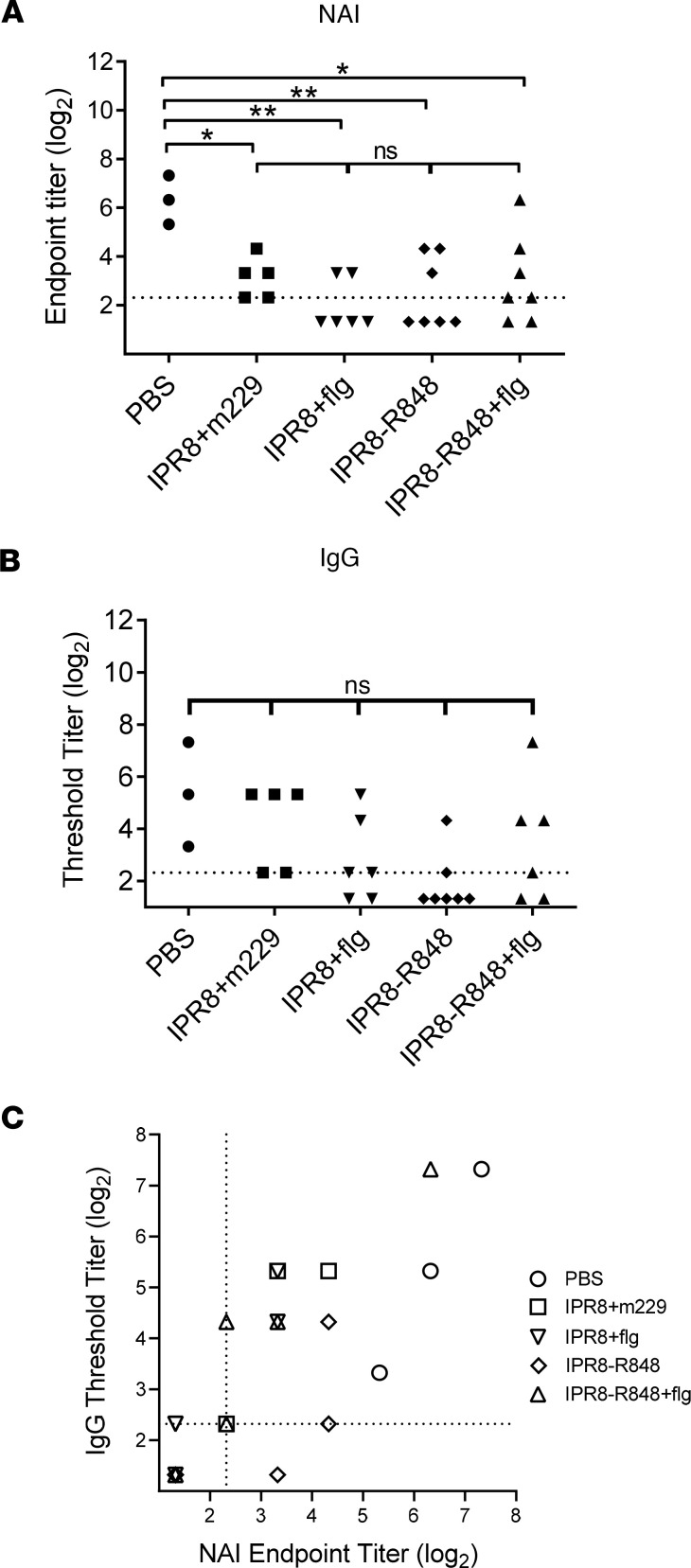
A portion of vaccinated newborns generate neutralizing antibody to NA in the respiratory tract after viral challenge. BAL samples were assessed 14 days p.c for NAI activity (**A**) and NA-specific IgG (**B**). Data from each readout for individual animals is shown in **C**. The dotted line shows the LOD for the assay. Statistical analysis was performed using 1-way ANOVA with Tukey’s correction for multiple comparisons. *P < 0.05, **P < 0.005.

Effective neutralization and clearance of influenza virus is dependent on antibody in the respiratory tract. Thus, we evaluated NAI antibody responses in the lungs of vaccinated newborns on d14 following challenge. In contrast to what was observed in our analysis of systemic antibody at this time point, vaccinated infants had lower levels of NAI antibody compared with nonvaccinated animals (Figure 4A). We had initially prioritized analysis by ELLA because of the limited sample. However, given the finding of reduced NAI antibody in the respiratory tract in the vaccinated groups, we performed an ELISA to quantify total NA-specific antibody. The trends were similar, although the decrease revealed by the ELISA readout did not reach statistical significance (Figure 4B). The values from the 2 readouts for the individual animals is shown in Figure 4C. This may suggest that the ELISA assay is less precise for detecting the lower levels of antibody present in the BAL. Nevertheless, these data reveal variable NAI antibody responses in the lungs of infected infants that were previously vaccinated with IPR8, resulting in significantly reduced amounts across the group as a whole.

### NA-specific antibodies do not emerge at later times in the vaccine response.

**Figure 5:**
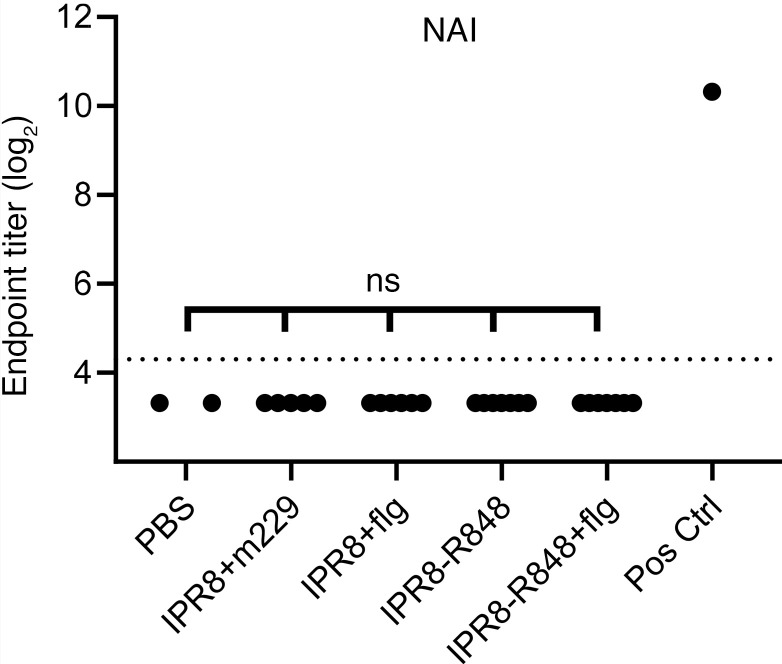
The inclusion of adjuvants that boost the overall response to PR8 does not induce detectable NA-specific antibodies on d100 following vaccination. Vaccinated and nonvaccinated newborn AGMs were evaluated by ELLA at approximately d100 following vaccination. The analysis included newborns vaccinated with IPR8+m229 (*n* = 5), IPR8+flg (*n* =6), IPR8-R848 (*n* =7), or IPR8-R848+flg (*n* = 7). Two animals were in the control group that received PBS. The data from an infected newborn run in parallel with the assay is shown as the positive control. The dotted line shows the LOD for the assay. Statistical analysis was performed using 1-way ANOVA with Tukey’s correction for multiple comparisons.

In our evaluation, we had also administered our experimental vaccines to a group of newborns that were not challenged; i.e., the antibody response was allowed to develop over a longer period of time. We used this cohort to test whether the failure to detect NA-specific antibody in vaccinated newborns on d10 p.b. was the result of assessment at a time point at which full maturation of the antibody response had not occurred. This could be the result of a difference in kinetics with regard to the differentiation of NA-specific activated B cells to antibody-secreting cells compared with more dominant responses. The presence of NAI antibody was evaluated in newborns on approximately d100 following initial vaccination. Animals received the vaccine on the same schedule as those tested above. We found no evidence of NAI antibody at this time point (Figure 5). As above, an infected newborn served as a positive control for the detection of antibody. These data add further support to the inability of the vaccine to induce NA-specific antibody responses.

## Discussion

NA is emerging as an attractive target for influenza vaccines, as antibodies against NA can be protective and often exhibit increased breadth of recognition across strains (13, 14, 17, 18). While a clearer picture of this response is developing in adults, our understanding of the ability of newborns to generate NA-specific antibodies, following either infection or vaccination, is highly limited. Here we probed this question using a newborn NHP model. We found that newborn animals make a robust antibody response to NA following infection and that these antibodies are highly capable of inhibiting enzymatic activity of the protein. In contrast, these responses were not detectable following vaccination with inactivated influenza virus, even in the presence of adjuvants that drive significantly higher total antibody responses to the virus (40, 41). Circulating NAI antibodies were efficiently generated following virus challenge of the vaccinated newborns, reaching circulating levels that were similar to those of nonvaccinated newborns. However, while systemic levels were similar, in the respiratory tract NAI antibody levels were reduced in vaccinated compared with nonvaccinated animals.

The newborn response to influenza virus infection and vaccination has primarily been studied in the mouse model (e.g., refs. 44–48). Deficits have been reported in both the CD4^+^ and CD8^+^ T cell compartments (44, 45) as well as the antibody response (47, 48). In addition, there is some evidence that the B cell repertoire of newborns is altered (49–52), leaving open the possibility of a divergent ability to respond to the NA antigen. Thus, it was crucial that the inherent ability of newborns to mount an antibody response to NA be evaluated. We initially employed what is arguably the most potent immune challenge, viral infection, finding that newborns were highly competent for production of antibodies that can recognize NA and inhibit its enzymatic activity. This result is promising, as previous human studies have demonstrated that NA-specific antibodies can contribute to protection (14). In addition to preventing the spread of new virions to neighboring cells, NA-specific antibodies can also facilitate clearance by promoting antibody-dependent cellular cytotoxicity (ADCC) (53).

It was perhaps not unexpected that vaccination did not induce detectable NAI antibody. In humans, the inactivated influenza virus vaccine is inconsistent in driving NA-specific antibody responses (54, 55). This is likely due, at least in part, to the failure to standardize NA content in vaccines and the lower level of NA compared with HA in the virion (56). With that said, we were a bit surprised that we did not detect NA-specific antibody in the newborns receiving the adjuvanted vaccines, given the significant increase in total influenza virus–specific IgG in these animals (40, 41). We acknowledge that we cannot rule out the possibility that some animals make low levels of antibody that were not detected in our analysis or did so transiently in a time period that was not assessed.

Administration of the current seasonal inactivated vaccine would be anticipated to leave naive infants without measurable NA-specific antibodies, similar to what we observed in our NHP study. The effect of these non-NA, influenza-specific antibodies on the ability to generate an NA-specific response following infection is of significance given the potential benefit these antibodies can provide (13, 14, 17, 18). We found that on d14 p.i., vaccinated newborn NHPs had circulating levels of NAI antibodies that were similar to those observed for non-vaccinated newborns. This result suggests that any preexisting influenza-specific antibody present as a result of vaccination did not impair the systemic NA-specific antibody response following homologous virus encounter. In contrast, vaccine-induced preexisting immunity appears to have had a regulatory effect on local generation of NAI antibody in the respiratory tract of a significant portion of newborns. Interestingly, a decreased antibody response in the lung on d14 following infection was not evident in our analysis of total PR8-specific IgG (40, 41), suggesting this may be specific to NA.

We propose that the reduced NA-specific antibody present in the lungs of vaccinated infants following infection could result from an impaired ability of vaccine-induced memory B cells to differentiate into antibody-secreting cells or a reduced capacity to mount a primary NA-specific response. What might be responsible for a lung-specific effect? Local responses in the lung following infection with influenza virus are associated with the formation of bronchus-associated lymphoid tissue (BALT) (57). Antigen acquisition in BALT can occur via M cells if BALT forms near the epithelial surface or through DC migration into BALT that forms deeper in the tissue (58). How DCs are directed to the draining lymph node versus BALT is not clear; however, their migration could impact the access of B cells to antigen. It is also possible that virus particles or viral antigens travel more efficiently to the lymph node, again allowing larger amounts of antigen for binding to B cells. It does not appear that BALT has afferent lymphatics, and thus viral antigen or antigen-bearing cells that enter the afferent lymphatics may be more likely to reach the draining lymph node. It is possible that the requirements for efficiently eliciting the less-dominant NA response are more stringent, and thus changes in antigen load or innate signals that are present in BALT versus the draining lymph node result in differential production of these antibodies.

In conclusion, we have shown that newborn AGMs generate antibodies that have NA-inhibiting activity following influenza virus infection. This differs from the response to vaccination, in which antibody to NA could not be detected on either d10 p.b. or approximately d100 following vaccination, even when adjuvants that significantly increased total influenza-specific IgG antibody were included in the vaccine (40, 41). The presence of non-NA antibodies to influenza virus did not impact the level of circulating antibodies to NA generated as a result of infection; however, their presence was associated with a decrease in NA antibodies in the lungs of a substantial portion of newborns. While the results from these studies demonstrate the ability of newborns to generate NAI antibodies, approaches designed to focus the response on this protein — e.g., increasing the dose of NA in current vaccines, use of recombinant proteins or NA-expressing DNA vaccines — will be required to elicit robust levels of these antibodies that can contribute to protection.

## Methods

### Animals.

AGM newborns used in this study were housed at the Vervet Research Colony at Wake Forest School of Medicine. Vaccinated newborns were raised in a nursery setting (36, 40, 41). Nonvaccinated newborns infected with virus were mother reared.

### Influenza A/Puerto Rico/8/34 (H1N1).

Influenza A/Puerto Rico/8/34 (H1N1) (PR8) virus stock for infection was grown and titered in fertilized chicken eggs essentially as described previously (59). Stocks were diluted in PBS, flash frozen, and stored at –80°C.

### Infection and sampling.

Four adult AGMs (6–9 years old) were sedated with 10–15 mg/kg ketamine. Four newborn animals (6–10 days old) were sedated with 2%–5% inhalant isoflurane. Adults received 5 × 10^9^ EID_50_ PR8 by combined intratracheal (i.t.) (1.0 mL) and i.n. (0.5 mL/nostril) routes. The dose was delivered equally between the i.n. and i.t. routes. Three newborns were administered at 1 × 10^9^ EID_50_, and 1 received 1 × 10^8^ EID_50_ (0.25 mL i.t. and 0.125 mL/nostril). No dose-related difference was present in the newborn animal that received the lower compared with those receiving the higher dose. Thus, these animals were pooled for the study. Blood was collected in sodium heparin tubes by venipuncture on d14 p.i. BAL was performed at necropsy (d14 p.i.) with 25 mL used for adults and 5 mL for infants. Samples were centrifuged to remove cellular material, and BSA was added to a final concentration of 0.5%.

### Vaccination.

Newborns were vaccinated with 45 μg of 0.74% formaldehyde-inactivated PR8 (IPR8) alone, IPR8 mixed with 10 μg flagellin (flg), IPR8 conjugated to R848 (IPR8-R848), IPR8-R848+flg, or IPR8 mixed with an inactive flagellin (m229) (60). All injections were delivered intramuscularly in the deltoid muscle (500 μL volume). Animals were boosted 21 days later. Nonvaccinated control animals received PBS. The R848-conjugated virus was prepared as previously described (40). Briefly, an amine derivative of R848 (hereafter referred to as R848) was linked to SM(PEG)_4_ by incubation in DMSO for 24 hours at 37°C. R848-SM(PEG)_4_ was then incubated with influenza virus (IPR8-R848). Unconjugated R848 was removed by extensive dialysis, followed by inactivation with 0.74% formaldehyde overnight at 37°C. Formaldehyde was removed by dialysis. Successful conjugation was assessed by differential stimulation of RAW264.7 cells by R848-conjugated versus nonconjugated vaccine (42).

Flagellin from *Salmonella enteritidis* was prepared as previously described (60). Briefly, *E*. *coli* BL21 (DE3) containing a pet29a::*fliC* encoding WT flagellin or the truncated pet29a::229 encoding only the biologically inactive hypervariable region of flagellin (60, 61) were grown and lysates prepared in 8 M urea. Proteins were purified on Ni-NTA agarose (QIAGEN) according to the manufacturer’s protocol. Endotoxin and nucleic acids were removed using an Acrodisc Mustang Q capsule (Pall). Purified proteins were extensively dialyzed against PBS.

### ELLA.

ELLA uses a reassortment H6N1 containing the HA (H6) gene from A/Turkey/Massachusetts/3740/1965 and the internal protein gene segments and NA from PR8 (34). This virus was provided by Maryna Eichelberger, Center for Biologics Evaluation and Research, US Food and Drug Administration (Bethesda, Maryland, USA). Virus was inactivated with β-propiolactone, aliquoted, and stored at –80°C. Prior to the assay, 96-well plates were coated overnight at 4°C with 100 μL fetuin diluted in PBS (25 μg/mL). The next day, the plate was washed 3 times with PBS + 0.05% Tween 20 (PBST). Plasma or BAL samples were heat inactivated (56°C for 45 minutes) and then serially diluted across the plate in PBS + 1% BSA + 0.1% Tween 20. Inactivated virus was added at a dilution previously determined to result in 90% maximal NA activity, and plates were incubated at 37°C for 18 hours. Plates were washed 6 times, with PBST and peanut agglutinin–HRP (PNA-HRP) diluted in PBS + 1% BSA (used at the highest dilution that gave the maximum signal when titrated on fully digested fetuin) added and incubated at room temperature for 1 hour. The plates were washed 3 times, developed with 3,3′,5,5′-tetramethylbenzidine (TMB; MilliporeSigma) for 20 minutes, stopped with 2N H_2_SO_4_, and read at 450 nm on a BioTek Elx800 Absorbance Microplate Reader. The starting dilution was 1:20 for plasma and 1:5 for BAL. The results were used to determine the 50% endpoint titer using the formula 100 × (OD_virus-only control_ – OD_test sample_)/OD_virus-only control_. Statistical analysis was performed using GraphPad Prism software on log_2_-transformed values. Results that were below the limit of detection were assigned a value 1 dilution greater than the initial tested (e.g., if 1:20 was the initial dilution, values were assigned as 1:10) for graphing and statistical purposes.

### Cell-based NA ELISA.

The ELISA was adapted from the protocol published by Wan et. al (33). Human embryonic kidney cells (293F, Gibco) grown to approximately 80% confluence in DMEM + GlutaMAX containing 7% FBS and were seeded into a 96-well round-bottom plate at 25,000 cells/well. Twenty-four hours later, cells were transfected with 0.2 μg per well of PR8 NA–expressing plasmid (pCAGGS-NA), provided by Jonathan Yewdell (NIH, Bethesda, Maryland, USA), using Lipofectamine 2000 in FreeStyle 293 expression medium with GlutaMAX (12338-018 Gibco). Cells were incubated for 5 hours at 37°C, medium was removed, and DMEM + GlutaMAX containing 7% FBS was added. Cells were cultured for an additional 48 hours to allow expression of NA, after which cells were fixed with 0.05% glutaraldehyde and washed with PBS. As a control for successful transfection, a parallel culture was harvested and expression of NA confirmed by flow cytometric analysis following staining with a monoclonal anti-influenza A virus NA antibody (clone NA2-1C1,NR-50239, BEI Resources). The fixed plates were blocked with DMEM+ GlutaMAX + 7% FBS for 1 hour at 37°C. Samples were then added for 1 hour at 37°C before being washed with PBST. Antibody was detected using anti-NHP IgG HRP (43R-IG020HRP, Fitzgerald). Plates were developed with TMB for 30 minutes and stopped with 2N H_2_SO_4_. Results that were below the limit of detection were assigned a value 1 dilution greater than the initial tested (e.g., if 1:20 was the initial dilution, values were assigned as 1:10) for graphing and statistical purposes.

### Statistics.

Significance was determined using an unpaired 2-tailed Student’s *t* test or 1-way ANOVA with Tukey’s correction for multiple comparisons as appropriate. All analyses were performed with GraphPad Prism software. A *P* value less than 0.05 was considered significant.

### Study approval.

All animal protocols were approved by the Institutional Animal Care and Use Committee of Wake Forest School of Medicine and adhere to the US Animal Welfare Act and Regulations.

## Author contributions

PKS designed and performed experiments, analyzed data, and wrote the initial draft of the manuscript. KFC performed experiments and analyzed data. BCH performed experiments. MAAM designed experiments and edited the manuscript. All authors contributed to editing of the final manuscript.

## Supplementary Material

Supplemental data

## Figures and Tables

**Table 1:**
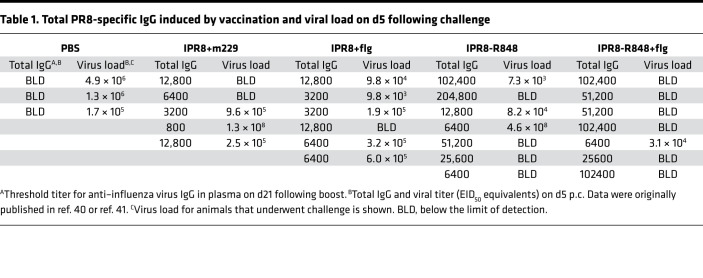
Total PR8-specific IgG induced by vaccination and viral load on d5 following challenge

## Version 1 12/17/2020

Electronic publication

## Version 2 12/02/2020

In-Press Preview
