# Supplementary material for: Neuraminidase-specific antibody responses are generated in naive and vaccinated newborn nonhuman primates following virus infection
Source: JCI Insight. 2020 Dec 17;5(24):e141655. doi: 10.1172/jci.insight.141655 (PMC7819742; doi:10.1172/jci.insight.141655)
Supplement: Supplemental data [file jciinsight-5-141655-s007.pdf]

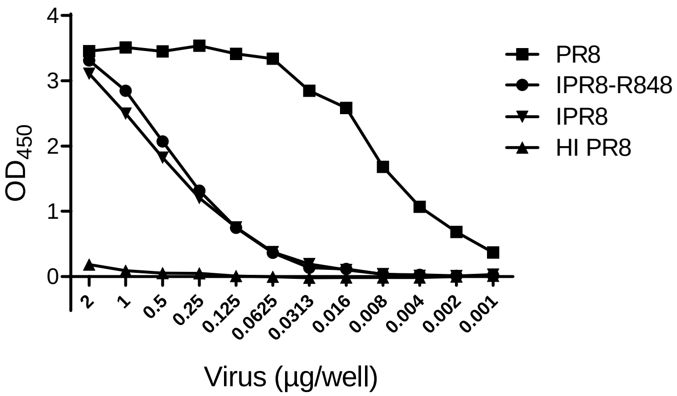

SF1. Formalin inactivated PR8 has reduced NA activity compared to live PR8. PR8, IPR8, IPR8-R848, and heat treated PR8 were tested for the level of NA activity using the ELLA assay. Heat inactivation was performed by placing virus at 56°C for 1 hour.
